# Profound loss of general knowledge in retrograde amnesia: evidence from an amnesic artist

**DOI:** 10.3389/fnhum.2014.00287

**Published:** 2014-05-06

**Authors:** Emma Gregory, Michael McCloskey, Barbara Landau

**Affiliations:** Department of Cognitive Science, Johns Hopkins UniversityBaltimore, MD, USA

**Keywords:** memory, general world knowledge, brain damage, retrograde amnesia, single case study

## Abstract

Studies of retrograde amnesia have focused on autobiographical memory, with fewer studies examining how non-autobiographical memory is affected. Those that have done so have focused primarily on memory for famous people and public events—relatively limited aspects of memory that are tied to learning during specific times of life and do not deeply tap into the rich and extensive knowledge structures that are developed over a lifetime. To assess whether retrograde amnesia can also cause impairments to other forms of general world knowledge, we explored losses across a broad range of knowledge domains in a newly-identified amnesic. LSJ is a professional artist, amateur musician and history buff with extensive bilateral medial temporal and left anterior temporal damage. We examined LSJ's knowledge across a range of everyday domains (e.g., sports) and domains for which she had premorbid expertise (e.g., famous paintings). Across all domains tested, LSJ showed losses of knowledge at a level of breadth and depth never before documented in retrograde amnesia. These results show that retrograde amnesia can involve broad and deep deficits across a range of general world knowledge domains. Thus, losses that have already been well-documented (famous people and public events) may severely underestimate the nature of human knowledge impairment that can occur in retrograde amnesia.

## Introduction

The study of amnesic individuals has contributed to the development of theories about normal and impaired memory. In particular, numerous studies of retrograde amnesia (impaired memory for previously-learned information) have explored losses of episodic (autobiographical) memory (e.g., Bayley et al., 2003; Rosenbaum et al., 2008) and in turn have led to specific proposals about how such information is encoded, retained, retrieved, and lost (e.g., Squire, 1992; Winocur and Moscovitch, 2011). Much less attention, both theoretical and empirical, has been paid to retrograde amnesia for another important type of memory content: general world knowledge. By general world knowledge, we mean knowledge that represents the rich and extensive sets of interconnected facts that are naturally acquired over a lifetime—facts such as the basic rules of baseball, the titles and creators of famous paintings, the companies represented by familiar logos, and the melodies associated with events such as birthdays and weddings. Understanding of how memory for our vast world knowledge is affected in retrograde amnesia is surprisingly limited, due in part to a restricted range of content tested. Previous studies have focused almost exclusively on memory for famous people and major public events—knowledge domains that may not be as entrenched as other aspects of our world knowledge. Without testing the limits of retrograde memory losses across many knowledge domains, we simply do not have a full understanding of how memory for non-episodic information may be affected by brain damage. Therefore, the aim of the present article is to begin filling in our understanding of the breadth and depth of losses possible in retrograde amnesia. To do so, we present a newly-identified amnesic, LSJ, and test her retrograde memory for general world knowledge across everyday knowledge domains as well as areas of pre-morbid expertise. Examining a broad range of knowledge domains, encompassing both everyday and expert knowledge, allows us to test the limits of non-episodic retrograde memory losses. Moreover, probing highly-overlearned everyday knowledge domains, and especially domains of personal expertise, allows us to ask whether significant memory loss may occur even in areas that might be the best cases for preservation.

Memory for general world knowledge, as we define it in this paper, can be contrasted with several other kinds of memory content, each of which has played a prominent role in theories concerning the structure of memory. Unlike episodic memory (which is tied to specific personal experiences, e.g., Tulving, 1972), general world knowledge is removed from the personal: An individual may not remember what happened at her 10th birthday party (episodic) yet still know perfectly well that “Happy Birthday” is the song people sing at birthday parties (general world knowledge). Whereas the former is experienced at just one point in time, the latter is part of a cultural experience that extends over a lifetime and, although it can be quite detailed, is also abstracted away from the space and time in which it was acquired. General world knowledge can also be contrasted with lexical semantic knowledge (sometimes called semantic or conceptual knowledge). The focus for studies of lexical semantic knowledge tends to be how individual words (and non-verbal content such as pictures or sounds) map onto meaning (e.g., Hodges et al., 1995; Schmolck et al., 2002). This type of knowledge is typically evaluated using a variety of tasks (e.g., reading aloud, lexical decision, picture categorization, copy drawing, see Patterson, 2007, for more examples), and is of particular interest in studies of individuals with semantic dementia, a neurodegenerative disorder that typically leads to impaired vocabulary and object concepts following damage to anterior temporal areas (e.g., Hodges et al., 1992; Bozeat et al., 2000; Lambon Ralph and Howard, 2000; Hodges and Patterson, 2007; Patterson et al., 2007). In contrast, general world knowledge (as we use the term) focuses on facts about the world, which can be expressed through language, but often as propositions that go beyond either the meanings of individual words or simple declarative referential statements such as “This is a baseball.” For example, someone may be able to name a picture of a baseball, spell the word, and read it aloud (intact lexical knowledge) but not know how many innings are played in a standard baseball game, or what city is the home of the Red Sox. Similarly, a person may be able to read and comprehend the word “government” but not know that the U.S. government has executive, legislative, and judicial branches, or that a Presidential election is held every 4 years.

Some studies of general world knowledge in retrograde amnesia have been reported, and have contributed to our understanding of memory for this distinct type of content. The focus, however, has largely been on whether memory losses can be characterized by a temporal gradient, such that there is greater sparing of remote than recent memories (e.g., Albert et al., 1979; Westmacott and Moscovitch, 2002) and the role of medial temporal structures in such temporally-graded memory (e.g. Kapur and Brooks, 1999; Smith and Squire, 2009). As a consequence, these studies have primarily tested knowledge that could be assumed to have been acquired at different points in time and experienced within a limited time period, allowing researchers to assess whether losses can be characterized by a temporal gradient. Two such areas of knowledge have been especially widely-used: famous people (names, faces, professions) and major public events (e.g., Reed and Squire, 1998; Steinvorth et al., 2005). For example, in a review 50 years of studies of retrograde memory deficits following temporal lesions, Lah and Miller (2008) analyzed 90 patients in 62 studies of retrograde memory testing autobiographical memories, memories for public events, or memories for famous people (for details see Table 1 in Lah and Miller, 2008). Remarkably, only 5 of the 62 studies tested an area of general world knowledge other than famous people or events, highlighting the heavy reliance on this circumscribed kind of knowledge in studies of retrograde amnesia.

Thus our understanding of how memory breaks down in retrograde amnesia has a significant gap: What consequences are there for the vast store of general world knowledge humans have acquired and used over a lifetime and in a wide variety of contexts, both formal and informal? We might expect this kind of knowledge to be highly resistant to disruption, by virtue of frequent exposure and repeated use across varied contexts. Alternatively, even this exceedingly well-learned and often-accessed knowledge may be vulnerable to disruption in retrograde amnesia, resulting in failure to remember even apparently obvious facts such as the number of holes on a professional golf course or what product Tony the Tiger represents.

Similarly, we can ask whether there is special protection for knowledge that represents personal areas of expertise—aspects of knowledge that are more tied to individual experiences and are a privileged part of a person's knowledge repertoire. Like areas of common knowledge, personal areas of expertise are well-learned, frequently accessed, and used in a variety of contexts. But domains of expertise differ from other domains of knowledge in that they may have additional personal significance, based on one's interest or motivation relating to the knowledge area, and they may be part of a more richly interconnected knowledge structure. Indeed, some have argued that general world knowledge representation may be different for domains of individual expertise than for domains assumed to be part of everyday world knowledge (e.g., Glaser and Chi, 1988). Specifically, knowledge in an area of expertise may be a distinct, organized body of knowledge such that it can be more readily retrieved and used than common general world knowledge (Glaser and Chi, 1988). Studies on loss and preservation of expert knowledge among amnesic individuals have reported mixed results, with expert knowledge sometimes preserved despite loss of autobiographical episodic memory (Tulving et al., 1988), and sometimes impaired along with episodic and non-expert memory (e.g., Yasuda et al., 1997). One recent study raises the possibility of preservation in an area of premorbid expertise (music) despite severe memory impairment for other kinds of knowledge (Finke et al., 2012). A former professional cellist, PM, became amnesic following encephalitis and bilateral damage to his medial temporal lobes. PM was presented with carefully constructed familiarity tests of retrograde (and anterograde) memory for classical compositions, and performed comparably to musician controls in discriminating targets from foils. In contrast, PM was reported to be impaired in recalling facts, including those in non-expert areas (e.g., rivers in his country or lyrics of well-known songs) and those in areas of expertise (e.g., recalling names of composers).^1^ However, aspects of the study design preclude firm conclusions about whether the preserved knowledge was due to its privileged domain or to question format.^2^ Moreover, in each of these studies “expert” knowledge was based on knowledge relating to one's profession. In this paper, we define personal expertise more broadly, as encompassing areas of knowledge that are not found in everyone but have personal significance for the individual as well as being are highly-learned, frequently accessed, and used across a range of contexts.

To summarize, we know little about the details of preservation and impairment in general world knowledge due to retrograde amnesia. Yet this type of knowledge is a central part of human cognition, representing a large swath of what we have learned over a lifetime. Pushing the boundaries regarding the extent and nature of memory loss within general world knowledge can help us understand how broad and deep retrograde amnesia can be, and whether there might be differences across domains that could plausibly have greater internal organization due to expertise. Moreover, understanding how this body of knowledge is compromised after brain damage can help us develop more articulated theories of memory content, including the question of whether general world knowledge can be considered distinct from those aspects of memory that have been more well-studied.

The present study represents a step in this direction by exploring retrograde memory across a range of general world knowledge domains in LSJ. LSJ was a professional illustrator, accomplished amateur musician, and history buff who contracted herpes simplex encephalitis, sustaining severe bilateral medial temporal lobe damage. High resolution magnetic resonance imaging revealed virtually no hippocampal tissue bilaterally, substantial tissue reduction in parahippocampal, entorhinal and perirhinal cortices, and severe damage to the left anterior portion of the lateral temporal lobe. LSJ exhibits profound anterograde amnesia (the inability to create new memories) as well as retrograde amnesia. In contrast, LSJ is largely intact with respect to general intellectual abilities, visual-spatial cognition, and vocabulary and other language functions (somewhat surprisingly given her anterior temporal damage).

In addition to examining how our vast general world knowledge—learned and used across a lifetime and in many contexts—can be disrupted in retrograde memory impairments, we examined potential differences in the extent of impairment between everyday knowledge and knowledge in areas of expertise. Because LSJ had premorbid expertise in several areas (art, music, history), her case afforded the opportunity to contrast multiple domains of both everyday knowledge and premorbid expertise. Specifically, we tested LSJ's memory associations in three areas of everyday knowledge (commercial logos, sports, everyday songs) and three areas of premorbid expertise (famous paintings, famous musical compositions, United States history).

We report that LSJ's severe retrograde amnesia extends across all knowledge domains tested, including not only everyday knowledge but also expertise she acquired in her professional training and career. The results suggest that the affected areas of the temporal lobe play an important role in storing and/or retrieving many different kinds of knowledge, including knowledge that would have been acquired and accessed across one's lifetime and in various contexts.

## Materials and methods

### Participants

All control participants provided written informed consent, and LSJ provided oral assent, with written consent provided by her legal guardian. The study protocol was approved by the Homewood Institutional Review Board at Johns Hopkins University.

#### Patient LSJ

LSJ is a 63-year-old woman with a B.F.A degree (Bachelor of Fine Arts, 16 years of education). She was a highly accomplished artist and illustrator, with an extensive portfolio that includes book illustrations, advertising illustrations for corporate clients, and illustrations for *New York Times* articles and *New Yorker* covers. In addition she was an amateur violist who played in orchestras and chamber groups in high school, college, and as an adult; she was a small-plane pilot with her own airplanes and airstrip; and she was a U.S. history enthusiast.

**Figure 1:**
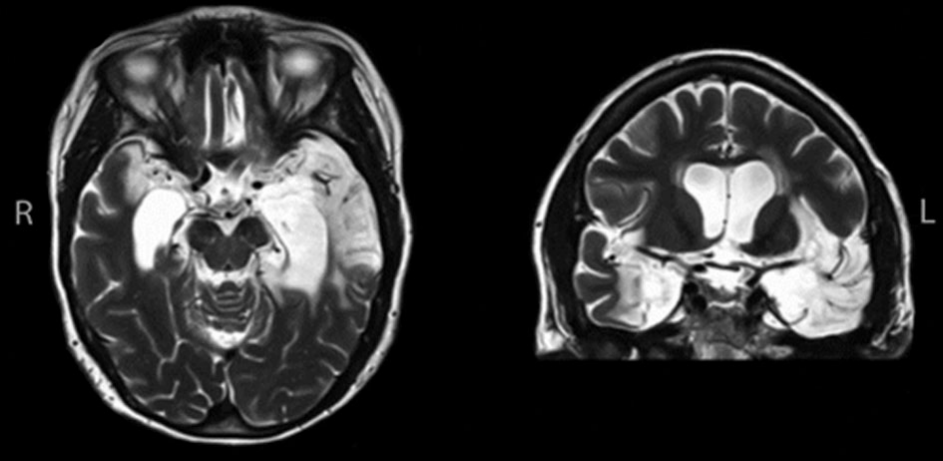
**High resolution magnetic resonance images showing the extent of temporal lobe damage in patient LSJ**. See text for description of lesion.

In December 2007, at age 57, LSJ contracted herpes simplex encephalitis, resulting in extensive brain damage (see Figure 1), including virtually complete destruction of the hippocampus bilaterally, bilateral medial temporal lobe damage, and anterior temporal damage more extensive on the left than on the right. Volumetric analyses of hippocampal and medial temporal lobe regions carried out on high-resolution magnetic resonance imaging data quantified these results: LSJ had bilateral reduction in hippocampal volume (approximately 96% loss on the left and 100% on the right), parahippocampal cortex (approximately 88% reduction on the left and 38% on the right), entorhinal cortex (approximately 100% loss on the left and 57% on the right), and perirhinal cortex (approximately 98% loss on the left and 50% on the right); see Schapiro et al. (2014), for detailed methods and results. In addition, whereas right-hemisphere damage was limited to the medial temporal lobe, left antero-lateral and ventral temporal regions also sustained severe damage, with obliteration of the temporal pole and some damage extending as far posterior as the mid-fusiform gyrus. Left insular and orbitofrontal cortex also showed evidence of damage.

***Neuropsychological assessment***. Neuropsychological assessment results are summarized in Table 1. On the Wechsler Memory Scale III (Wechsler, 1997) LSJ showed catastrophic impairment on all tasks requiring retention of information for more than a few seconds, indicating severe anterograde memory impairment. Her performance was also impaired, although less drastically so, on the working memory tasks. She drew an excellent copy of the Rey-Osterrieth complex figure (Osterrieth, 1944), scoring 34/36, but when asked to draw the figure from memory 10 min later she scored 0/36, drawing a simple shape with little resemblance to the stimulus figure. On the Warrington Recognition Memory Test (Warrington, 1984), which probes anterograde memory for faces and words, she performed at chance.

**Table 1:** **Neuropsychological profile of patient LSJ**.

**Test**	**Score**	**Relative to controls**
**WECHSLER ADULT INTELLIGENCE SCALE IV (WAIS-IV, Wechsler, 2008)**		
Full Scale	92	30th percentile
Verbal comprehension	96	39th percentile
Perceptual reasoning	104	55th percentile
Working memory	83	13th percentile
Processing Speed	86	18th percentile
**WECHSLER MEMORY SCALE III (WMS-III)**		
Auditory immediate	56	0.2 Percentile; impaired
Visual immediate	57	0.2 Percentile; impaired
Immediate memory	47	<0.1 Percentile; impaired
Auditory delayed	58	0.3 Percentile; impaired
Visual delayed	56	0.2 Percentile; impaired
Auditory recognition delayed	55	0.1 Percentile; impaired
General memory	47	<0.1 Percentile; impaired
Working memory	76	5th percentile; impaired
**WARRINGTON RECOGNITION MEMORY TEST**		
Words (/50)	26	Impaired
Faces (/50)	28	Impaired
**REY-OSTERRIETH COMPLEX FIGURE**		
Copy (/36)	34	
Delayed recall (10 min, /36)	0	Impaired
Boston naming test (/60)	49	Normal adult 50–59 years old: mean = 55.8, *SD* = 2.6, range = 49–59
**WIDE RANGE ACHIEVEMENT TEST**		
Reading	103 (58th percentile)	Average
Spelling	102 (55th percentile)	Average
Peabody picture vocabulary test—revised (PPVT)	97 (42nd percentile)	Average
**VISUAL OBJECT AND SPACE PERCEPTION BATTERY (VOSP)**		
Screening test (/20)	20	Pass
Incomplete letters (/20)	20	Pass
Silhouettes (/20)	16	Pass
Progressive silhouettes (High Score = 2)	10	Pass
Dot counting (/10)	10	Pass
Position discrmination (/20)	20	Pass
Number location (/10)	9	Pass
Cube analysis (/10)	9	Pass
**RECOGNITION OF LINE DRAWING OBJECTS FROM DIFFERENT PERSPECTIVES**		
Canonical viewpoint (/39)	38	Average (Control mean/40 = 39.6; *SD* = 0.8; range = 38–40; reported in Landau et al., 2006)
Unusual viewpoint (/40)	32	Average (Control mean = 36.5; *SD* = 2.7; range = 30–40; reported in Landau et al., 2006)
Benton facial recognition test	46	Normal range = 41–54
Recognition of famous landmarks (/26)	2	Impaired (Control mean = 18.2; *SD* = 4.5; reported in Duchaine et al., 2003)
Recognition of famous faces (/60)	2	Impaired (Control mean = 52.6; *SD* = 5.2; reported in Duchaine et al., 2007)
**MONTREAL BATTERY OF EVALUATION OF AMUSIA (MBEA)**		
Scale	25	Pass
Contour	25	Pass
Interval	27	Pass
Rhythm	26	Pass
Meter	28	Pass
Incidental	16	Fail

In sharp contrast, LSJ scored in the normal range on tests of vocabulary (Peabody Picture Vocabulary Test-Revised, Dunn and Dunn, 1981), and single-word reading and spelling (Wide Range Achievement Test III, Wilkinson, 1993). On the Boston Naming Test (Kaplan et al., 1983) she scored at the low end of the normal range, and on the vocabulary subtest of the Wechsler Adult Intelligence Scale IV, LSJ was slightly above average (scaled score of 11). LSJ's visual-spatial abilities also appear to be spared, as indicated by her normal performance on the Rey-Osterrieth figure copy and the Visual and Object Space Perception Battery (Warrington and James, 1991). On a test of object recognition in which she named pictures of objects shown in typical and unusual perspectives (Landau et al., 2006), LSJ's performance was within the range of normal adults, regardless of viewpoint. In the realm of music perception, LSJ showed normal performance on the Montreal Battery of Evaluation of Amusia (Peretz et al., 2003), except on an incidental memory subtest.

LSJ's intact skills are also attested by her preserved ability to construct new pieces of art. With regard to medium, LSJ's pre-illness art involved drawing with pencil and pen as well as painting with watercolor; in her post-illness art she almost exclusively draws, though she is happy to paint with watercolor when asked. Her art retains many of the same qualities and is recognizably hers. Unlike her pre-illness art, LSJ's post-illness art is often organized through images integrated with word puzzles, underscoring her intact word knowledge.

In addition to art, LSJ's pre-illness skills are evident in the realm of music: when presented with novel sheet music for the viola, LSJ can sight read and play quite well. Yet more impressive is her musical expression, which is emotive and includes improvisations that fit with the style of the music being played.

Generally, LSJ presents as incredibly cheerful, especially if she has the opportunity to do something she enjoys, such as creating illustrations, playing her viola, or discussing art, music, flying, or word puzzles. She converses fluently and enthusiastically, and spends much of her time creating drawings and word puzzles.

***Retrograde memory assessment***. In probing LSJ's retrograde memory, we first examined two domains typically tested in studies of retrograde amnesia: autobiographical memory and famous faces.

In three autobiographical interviews totaling 90 min, LSJ was asked about her life prior to her illness. Modeled after the Autobiographical Memory Interview (Kopelman et al., 1989), the interviews included open-ended questions, probes about 15 life events (e.g., attending a birthday party, graduating from high school) from various life periods (early childhood, teenage years, young adulthood, middle adulthood), and questions about personal semantics (i.e., facts about herself, including family, schooling, jobs, interests). These extensive interviews failed to elicit recall of a single specific episode from LSJ's life. Typically, she responded to probes with vague, generic statements (e.g., “I remember that I enjoyed people” for the question *Do you remember going to a birthday party?*). LSJ was able to recall some personal semantic information (e.g., her parents' professions, the name of her elementary school, the first musical instrument she played), but her performance was characterized by lack of detail, and major knowledge gaps. For example, she expressed uncertainty as to whether she had ever been married, despite having been married for a period of approximately 10 years prior to her illness. No evidence of temporal gradients was evident in any of the results.

On the famous faces test developed by Duchaine et al. (2007), Duchaine et al. reported that older adult control participants (*N* = 8; mean age 63.9 years) named an average of 53 of the 60 faces (88%). LSJ was able to name only 2 of the faces (3%). To evaluate whether LSJ's performance differed from that of Duchaine et al.'s control's group, we used modified *t* tests that were developed to compare an individual's performance to that of a small sample (Crawford and Howell, 1998; Crawford and Garthwaite, 2002; Crawford et al., 2010). These statistics are between-group *t* tests which treat the patient (here LSJ) as a group of *N* = 1. The results revealed that LSJ's performance was significantly worse than that of the controls for the famous faces test, *t*(7) = 5.2, *p* < 0.001.

#### Control participants

Control participants for the everyday knowledge tasks and the United States history tasks were 10 neurologically intact individuals (5 female) matched in age and education to LSJ. The control participants' mean age was 62 (*SD* = 4.6; range = 55.5–69.1), their mean years of education was 17 (*SD* = 1.4; range = 16–20), and no participant had more than master's degrees. No control participant reported brain injury or damage, neurological disorder, learning disability, psychiatric disorder, drug or alcohol abuse, vision or hearing impairment, or medications that would affect cognitive function.

For the painting and classical music tasks, we recruited and tested controls with similar personal expertise to LSJ in art or music, respectively. Controls for the famous painting tasks (*N* = 10; mean age = 63, *SD* = 6.1, range 55.2–71.8; 7 female) had a B.F.A degree, B.A. degree in studio art, or the equivalent, and no more than a master's degree. Some of the participants, like LSJ, were professional artists. Controls for the classical music task (*N* = 9; mean age = 64, *SD* = 4.3, range 58–69; 8 female) had 8 or more years of training or experience in playing a musical instrument; none had degrees in music.

### Design and procedure: memory tests

The autobiographical memory and famous faces tasks demonstrate that LSJ shows profound retrograde memory impairment on the sorts of test typically used to assess retrograde amnesia. To determine whether and to what extent retrograde amnesia may affect broad general world knowledge, we tested LSJ's retrograde memory in six domains: three everyday knowledge domains (commercial logos, sports, everyday songs) and three areas in which she had premorbid expertise (art, classical music, United States history). Unlike the typically-examined general world knowledge domains of famous people and events, these areas involve knowledge that is encountered repeatedly across one's life time and in many contexts. For each domain, both a recall task and a recognition task were created, and each task was administered to LSJ twice, with two to thirteen months between administrations. In each domain the recall task was administered prior to the recognition task. For both the everyday knowledge and expert knowledge domains, we varied the stimulus-response properties such that both had tests with a visual stimulus-name response pairing (commercial logos, famous paintings), an auditory stimulus-name response pairing (everyday songs, famous musical compositions) and a verbal question-verbal response pairing (sports, U.S. history). Age- and education-matched control participants were tested with a single administration of each task.

#### Everyday knowledge domains

***Commercial logos***. Stimuli were 61 familiar logos for companies, products or organizations (e.g., the McDonald's golden arches logo; see Table S1). The logos were created between 1863 and 2004 (6 before 1950, 6 in the 1950s, 10 in the 1960s, 11 in the 1970s, 12 in the 1980s, 10 in the 1990s, and 6 in the 2000s), and all were in common use within the United States during LSJ's adult life. In the recall task, the logos were presented one at a time on a computer screen, and the participant was asked to name the company, organization or product. In the forced-choice recognition test the logo was presented with three alternatives: the name of the actual company/organization, a plausible alternative and an implausible alternative (e.g., McDonald's, Mrs. Fields, Mitsubishi). The plausible alternative was a company of the same category (e.g., Corvette for the Audi logo) or a company whose name was related to the logo (e.g., Applejacks for the Apple logo, which is a picture of an apple).

***Everyday songs***. Stimuli were brief (5–14 s) instrumental auditory clips of 20 musical pieces strongly associated with particular events or occasions (e.g., *Auld Lang Syne, We Wish You a Merry Christmas, Pomp, and Circumstance, Wedding March, Take Me Out to the Ballgame*; see Table S2). In the recall task the participant was asked to name the event or occasion for each musical excerpt. In the recognition task the participant chose from names of three alternatives displayed on a computer screen while the clip was played (e.g., graduation, wedding, and Olympics for *Pomp and Circumstance*).

***Sports***. Stimuli for recall and three-alternative forced-choice tasks included 55 questions probing basic knowledge about sports, including questions about rules, equipment, playing locations, professional teams, and professional athletes (e.g., *What U.S. city do the Red Sox come from?*; *What two seasons do the Olympics take place in?; In baseball, which player throws the ball to the batter?*; *What sport was Muhammad Ali famous for?; How many points is a soccer goal worth?*; See Table S3). The questions collectively were posed about 14 sports, and four of the questions referred to a picture that was displayed while the question was read (e.g., a picture of a player in a baseball uniform for the question, *What sport does this athlete play?*).

#### Areas of premorbid expertise

***Art***. LSJ was a professional illustrator with considerable academic training in art history, and her family confirmed that she was premorbidly very knowledgeable about Western art. This task tested her ability to identify the artist for famous works of Western art (e.g., Van Gogh's *Starry Night*, Botticelli's *Birth of Venus*; see Table S4). Stimuli were 63 famous paintings displayed on a computer screen; 20 of the paintings had been selected by LSJ's sister and mother as family favorites. In the recall task, the participant was asked to name the artist, whereas in the recognition task the painting was presented with three alternatives: the actual artist, a plausible alternative (based on style and time period), and an implausible alternative.

***Classical music***. LSJ has played the viola since childhood, and she has performed in amateur orchestral and chamber groups in high school, college, and thereafter. LSJ's sister, a cellist who often played with her, reported that premorbidly LSJ had substantial knowledge of classical music. Her knowledge for musical compositions was tested in two classical music tasks. Stimuli were 61 brief (4–20 s) clips of instrumental classical music pieces (e.g., Beethoven's Fifth Symphony, Mozart's Eine Kleine Nachtmusik; see Table S5) composed between the 1660s and the 1960s. The 61 pieces included 35 different composers (e.g., Bach, Brahms, Beethoven, Mozart, Shostakovich, Gershwin, Glass), with each composer represented by one to six pieces. In the recall task, the participant was asked to name the composer; in the recognition task, three alternatives were presented while the clip was played: the actual composer, a plausible alternative (based on style and time period), and an implausible alternative (e.g., Beethoven, Mendelssohn, and Gershwin for Beethoven's Fifth).

***United states history***. LSJ's family described her as a history buff, and reported that she was knowledgeable about U.S. history and government. Stimuli for the recall test were 46 questions concerning U.S. history and government selected and adapted from a test administered by the U.S. Citizenship and Immigration Services to individuals seeking to become naturalized citizens (e.g., *What is the capital of your state?*; *What are the two major political parties in the United States?; Who did the United States fight in World War II?*; *What are the two parts of the US Congress?*; see Table S6). The recognition task probed knowledge of U.S. Presidents. Stimuli were the names of the 43 Presidents and 43 other famous male figures in U.S. history (e.g., Oliver Wendell Holmes, John Wilkes Booth, Thomas Edison, Benjamin Franklin, Charles Lindbergh, Robert E. Lee; see Table S7). The 86 names were presented one a time in random order on a computer screen, and the participant was asked to indicate whether the person was ever a U.S. President.

## Results

For each knowledge domain, we evaluated LSJ's performance relative to controls using modified *t* tests designed to compare an individual's performance to that of a small sample (Crawford and Howell, 1998; Crawford and Garthwaite, 2002; Crawford et al., 2010).

### Everyday knowledge domains

#### Commercial logos

For the recall task, mean performance for control participants was 45/61 (74%) correct (*SD* = 7.8; range 31–54). In contrast, LSJ was able to name only 6 (10%) and 5 (8%) of the companies on the two administrations of the test. The LSJ-control differences were highly reliable, *t*(9) = −7.76 and −7.07, for the first and second administrations, respectively, *p*s < 0.001.

In the recognition task, mean control performance was 57/61 (93%) correct (*SD* = 2.8; range 52–60), but LSJ's accuracy was only 36/61 (59%) and 34/61 (56%) on the two administrations. Her performance, although above chance, was significantly worse than that of the controls, *t*(9) = −4.73 and −4.85, *p*s < 0.001.

#### Everyday songs

In the recall task, mean control performance was 18/20 (90%) correct (*SD* = 1.5; range 16–20), whereas LSJ was correct for only 4/20 (20%) and 7/20 (35%) of the items on the two administrations, *t*(9) = −8.99 and −7.04, *p*s < 0.001. Similarly, in the recognition task, controls were nearly perfect (mean = 19.9/20, *SD* = 0.3; range 19–20), but LSJ scored only 12/20 (60%) and 13/20 (65%), *t*(9) = −20.82 and −23.84, *p*s < 0.001. LSJ's poor performance is especially striking in light of the fact that the music-event associations are well-known by normal individuals, as indicated by the near-ceiling control performance.

#### Sports

In the recall task, controls averaged 50/55 (91%) correct (*SD* = 4.4; range 43–55), whereas LSJ scored only 16/55 (29%) and 18/55 (33%), *t*(9) = −7.26 and −6.83, *p*s < 0.001. On the recognition test, the control mean was 54/55 (98%) correct (*SD* = 1.2; range = 51–55), but LSJ was correct for only 31/55 (56%) and 32/55 (58%) of the items in the two administrations, *t*(9) = −17.54 and −16.78, *p*s < 0.001.

### Areas of premorbid expertise

#### Art

In the recall task, the art-knowledgeable control participants named the artist correctly for a mean of 45/63 (71%) of the paintings (*SD* = 8.6; range 27–57). In contrast, LSJ was correct for only 1 (2%) and 2 (4%) of the paintings, naming Da Vinci as the painter of *Mona Lisa* twice and *The Last Supper* once, *t*(9) = −4.81 and −4.70, *p*s < 0.001.

In the forced-choice recognition task, the art-knowledgeable controls averaged 58/63 (92%) correct (*SD* = 2.9; range 54–62), whereas LSJ chose the correct artist for only 31/63 (49%) and 21/63 (33%) of the items on the two administrations of the task, *t*(9) = −8.98 and −12.25, *p*s < 0.001.

#### Classical music

In the recall task the music-knowledgeable controls named the composer on an average of 26 of the 61 items (43%; *SD* = 11.2, range 5–47). LSJ, however, was unable to name any of the composers correctly on either administration of the task, *t*(8) = −2.21, *p* < 0.03. On the recognition task the controls averaged 45/61 (74%) correct (*SD* = 7.0, range 30–56), but LSJ scored only 20/61 (33%) and 24/61 (39%) on the two administrations, *t*(8) = −3.36 and −2.82, *p* < 0.02. In fact LSJ's recognition performance was at chance.

#### United states history

Whereas control participants averaged 42/46 (91%) correct (*SD* = 1.0; range 40–43), LSJ responded correctly for only 18.5 (40%) and 19.5 (42%) of the items in the two administrations (fractional scores reflect partial credit), *t*(9) = −22.2 and −21.3, *p*s < 0.001.

A similar result was obtained in the U.S. Presidents yes-no recognition test. Control participants averaged 83/86 (97%) correct. In contrast, LSJ scored 59/86 (69%) and 65/86 (76%) correct on the two administrations, *t*(9) = −16.8 and −12.6, *p* < 0.001. LSJ's misses (‘no’ responses to Presidents) included both early (e.g., Polk, Harrison) and more recent Presidents (e.g., Clinton, Carter, Obama).

### Summary of results

**Figure 2:**
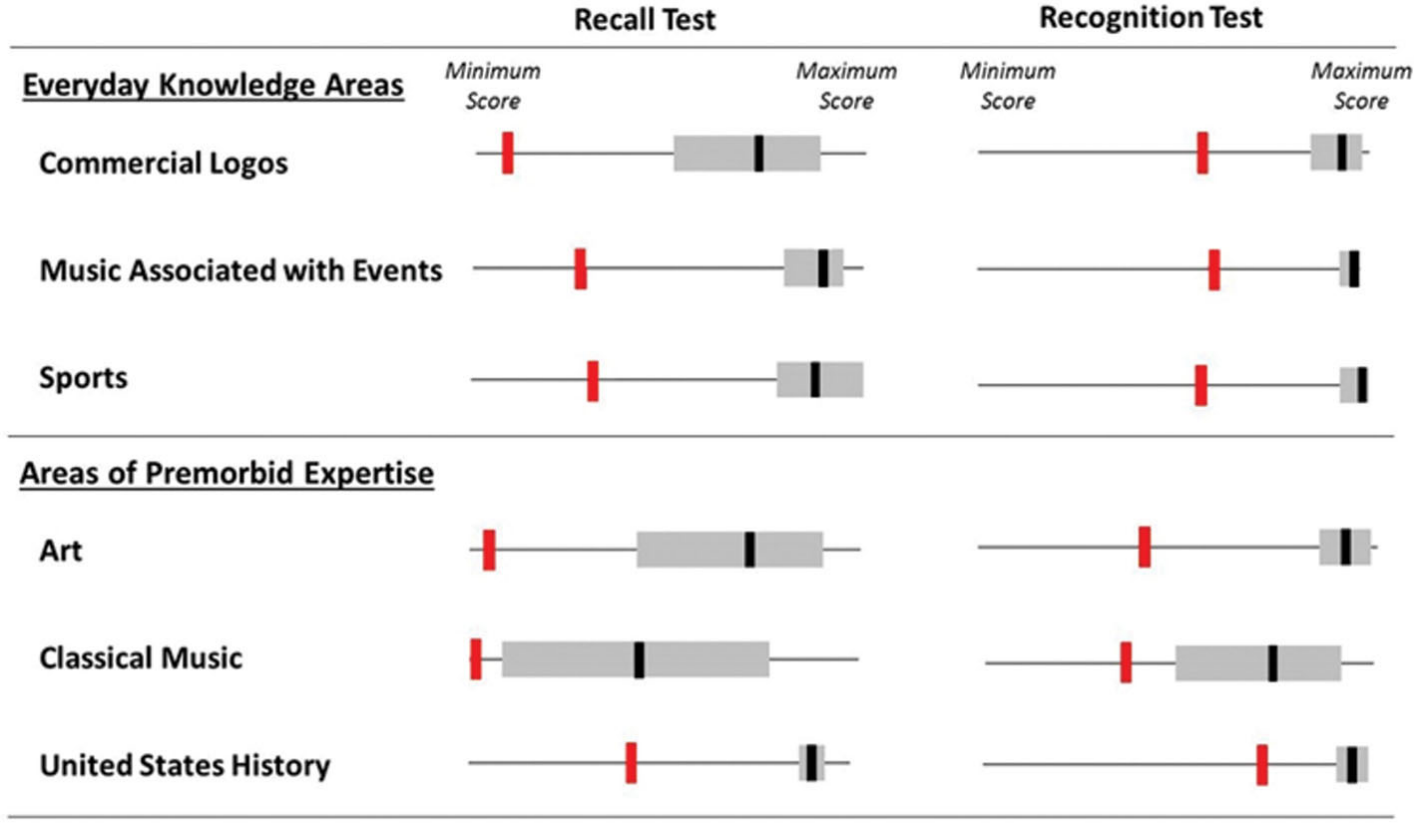
**LSJ's and controls' performance on everyday and expert knowledge domain recognition and recall tests**. The red bar represents LSJ's average score across two administrations of a test, the gray rectangle represents the control range, and the black bar represents the controls' mean score.

Figure 2 summarizes the results for the three everyday knowledge and three expert knowledge domains. As the figure makes clear, LSJ was profoundly impaired on all tasks, scoring significantly more poorly than controls, and indeed outside the control range.

## Discussion

To explore the extent to which general world knowledge can be impaired, we tested LSJ on a sampling of knowledge areas that, relative to previous studies of general world knowledge, was both larger and more varied with regard to the time and context in which one would be exposed to the information. Our findings suggest that retrograde memory deficits can extend across a broad set of domains involving knowledge encountered repeatedly over a lifetime and in a wide variety of contexts. Additionally, these domains included both everyday knowledge and premorbid expertise. LSJ's memory impairment included failure to remember everyday knowledge including famous people as well as commercial logos, everyday songs, and commonly known facts about sports. Her failures in areas of premorbid expertise included memory for works of art, classical music, and a wide range of facts about history and government. The content in these domains is likely to have been acquired over a wide timespan in LSJ's life, and in a variety of contexts including both formal and informal settings for learning. Together with the location of her brain damage, LSJ's performance supports the idea that the temporal lobes may be vital for storing many domains of world knowledge. The results also push the boundaries of what we know about the retrograde memory losses possible after temporal-lobe damage, establishing that losses can occur across a broad range of domains, even those representing pre-morbid expertise. This finding goes far beyond what has been shown in prior studies, or what would have been shown if we had only assessed LSJ's memory for famous people and major public events.

The breadth of LSJ's impairment raises several questions about the nature of memory for general world knowledge and its supporting neural structures. First, LSJ's impairment of general world knowledge is particularly striking in the context of her spared vocabulary. Along with an overall IQ within the normal range and high levels of performance on tests of perceptual and spatial abilities, LSJ performed in the normal range on standardized vocabulary tests, suggesting that much of her vocabulary knowledge is preserved. Her performance contrasts with that of semantic dementia patients, who have impaired lexical semantic knowledge, and accordingly perform in the atypical range on tests of vocabulary knowledge (e.g., Hodges et al., 1992). LSJ's largely intact vocabulary despite broad deficits in world knowledge supports the idea that these two aspects of memory represent different kinds of knowledge.

Second, while our findings establish LSJ's loss of retrograde general world knowledge, they also raise the question of whether her impairment reflects loss of memory for association between items or loss of familiarity for single items. The studies of retrograde amnesia that have examined memory for both item familiarity and for recall of associations (e.g., Reed and Squire, 1998; Bright et al., 2006; see Lah and Miller 2008 for a review of studies of retrograde memory) have shown mixed results, with item familiarity sometimes impaired along with recall (e.g., Eslinger, 1998) and sometimes spared despite impaired recall (e.g., Cipolotti et al., 2001). Our studies focus on LSJ's knowledge of associations between images and names, sounds and names, and questions and answers; but failure with these stimuli could stem from forgetting of items or associations. Further studies will be needed to better understand this particular dimension of LSJ's losses of general world knowledge.

Third, LSJ's case raises the question of which specific areas—the hippocampus proper, non-hippocampal medial temporal structures, or lateral temporal areas—are responsible for her extensive retrograde amnesia for general world knowledge (see Kopelman et al., 2003; Bright et al., 2006; for consideration of additional areas, i.e., frontal areas, thalamic regions). Currently, there is evidence showing impaired retrograde memory for general world knowledge in patients with selective damage in each of these regions. For example, there is evidence for impairment using tests of famous people and public events in patients whose damage is limited to the hippocampus (Rempel-Clower et al., 1996). Further evidence using tests of news events confirms impaired knowledge in patients with damage limited to the hippocampus as well as patients with damage to both hippocampus and other medial temporal structures, with more severe impairment for the latter (Bayley et al., 2006). There is also evidence of impairment on tests of famous people and scenes of famous news events in patients with damage limited to anterior lateral temporal areas (e.g., Kapur et al., 1992). These results, along with LSJ's, suggest that memory for general world knowledge may rely on multiple areas in the medial and lateral temporal lobe.

Although the merits of case-study methods have been debated at length in cognitive neuroscience (e.g., Caramazza and McCloskey, 1988; Kosslyn and Intriligator, 1992) it is widely acknowledged that single-patient studies have been enormously valuable in the realm of learning and memory. In particular, these cases have made major contributions to our knowledge about learning and memory at both cognitive and neural levels, and indeed have played a very significant role in shaping theories, questions, and research agendas (e.g., Scoville and Milner, 1957; Gabrieli et al., 1988; Squire and Knowlton, 1995; Chun and Phelps, 1999; Bartsch et al., 2011). The case of LSJ suggests that temporal-lobe damage can result in across-the-board memory losses, with the strongest possibility being that memory for virtually all domains of general world knowledge can be severely compromised. At the same time, it is possible that future cases will show that LSJ's losses are atypical, that is, unusually broad, even for patients with similar extensive damage. Many or even most patients with comparable brain damage may show distinctly different patterns of loss from that of LSJ, for example, patterns that reflect uneven distribution of losses across knowledge domains or highly selective losses across domains. In order to determine whether and how such profiles of memory loss might follow from particular patterns of damage, future research will need to include careful and extensive studies of memory loss across many knowledge domains. Our study of LSJ offers a model for such studies in going beyond the predominant current strategy of examining retrograde semantic losses in only a few rather limited domains.

In conclusion, our study presents what we believe to be the first in-depth examination of retrograde memory loss for a broad range of general world knowledge following severe temporal lobe damage. The findings show that such loss can be profound—certainly much broader and deeper than has been indicated through studies limited to content in the domains that are typically tested. Continued systematic and detailed probing of memory for general world knowledge in patients sustaining temporal lobe damage should lead to more fully articulated theories of this type of memory, which can then be used to better understand the neural and functional bases for memory deficits.

### Conflict of interest statement

The authors declare that the research was conducted in the absence of any commercial or financial relationships that could be construed as a potential conflict of interest.

## Footnotes

^1^These results are based on Finke et al. (2012) brief summary, with examples, of PM's performance on these tasks. The authors do not describe the methods or results for these tasks in detail.

^2^These tasks, unlike the tests of musical compositions that examine familiarity-based (recognition) memory, test memory for recollecting information. Thus, PM's preservation in an area of expertise is limited to familiarity-based memory in contrast to recollecting information, the type of memory more typically evaluated in studies of retrograde amnesia (e.g., Reed and Squire, 1998; Tulving et al., 1988). Moreover, given that non-expert retrograde memory was not tested using familiarity judgments, it remains unknown whether the preserved memory was specific to an area of expertise or was driven by a residual sense of familiarity as opposed to the ability to recollect information.
