# Supplementary material for: Profound loss of general knowledge in retrograde amnesia: evidence from an amnesic artist
Source: Front Hum Neurosci. 2014 May 6;8:287. doi: 10.3389/fnhum.2014.00287 (PMC4018544; doi:10.3389/fnhum.2014.00287)
Supplement: Supplementary file 1 [file DataSheet1.PDF]

Supplementary Material: Tables S1-S7

Table S1. Stimulus Items: Commercial Logos Recall and Recognition Test

Logo	Recognition Test Distractors
Chili's	Dr. Pepper, Old Spice
McDonald's	Mrs. Fields, Mitsubishi
Audi	Corvette, Rolex
Lexus	Lamborghini, Lacoste
WWF	Animal Planet, Air China
Aunt Jemima (older version, 1960s)	Mrs. Butterworth, Kentucky Fried Chicken
NBC (newer version, 1986)	ABC, GQ
Windows	Verizon, Oldsmobile
Girlscouts	Campfire Girls, Betty Crocker
Corvette	Cadillac, Chili's
Shell	Hamilton Beach, Lucky Charms
Red Cross	Doctors Without Borders, Texaco
Rolex	Cartier, Honey Nut Cheerios
Olympics Games	World Cup, CBS
Wendy's	Dairy Queen, Kool Aid
Apple (newer version, 2003)	Apple Jacks, McDonald's
Adidas	Nike, Yankees
Mercedes	Ferrari, Greyhound
Lucky Charms	Lucky Strike, Playboy
Ralph Lauren	Lacoste, John Deere
Bell Telephone	Taco Bell, Michelin
Cadillac (newer version, 2002)	Porsche, Bell Telephone
Cheetos	Cheez Doodles, Animal Planet
Mazda	Mercury, Mrs.Fields
Kentucky Fried Chicken (KFC)	Long John Silver's, Mr.Clean
Frosted Flakes	Rice Krispies, Cheetos
Honda	Hummer, Honeycomb
American Airlines	AirTran Airways, Auntie Anne's
Chevrolet	Mazda, Starbucks
Gerber	Enfamil, United Way
Oldsmobile	Pontiac, Olympics
Mustang	Dodge Charger, World Cup
NY Yankees	Mets, New York and Company
Michelin	Bridgestone, Honda
Pontiac	Mitsubishi, Shell
Texaco	Toyota, Toshiba
John Deere	International Harvester, Deer Park
NBA	MLB, CNN
Pepsi	Coke, Frosted Flakes

Cheerios	Honeycomb, Burt's Bees
Starbucks	Dunkin' Donuts, Aunt Jemima
Cadillac (older version, 1957)	Mercedes, American Airline
Apple (older version, 1976)	Applebee's, ABC
Taco Bell	Pacific Bell, Quicktime
Aunt Jemima (newer version, 1989)	Betty Crocker, Girlscouts
United Way	Allstate, Wendy's
Nike	Reebok, Chevrolet
Quicktime	Quicken, Dairy Queen
Pillsbury	Nestlé, Pepsi
Greyhound	K9 Advantix, Lexus
Kool-Aid	Juicy Juice, Red Cross
Air Jordan	NBA, Ralph Lauren
Ferrari	Mustang, Pillsbury
CBS	NBC, WWF
Toyota	Acura, Yamaha
NBC (older version, 1962)	CNN, MLB
Mr.Clean	Benjamin Moore, Dr. Pepper
Allstate	State Farm, Gerber
Playboy	GQ, Gymboree
Acura	Audi, AirJordan
YAMAHA	Steinway, Windows

Table S2. Stimulus Items: Music Associated with Events Recall and Recognition Tests

**Song**	**Event or Occasion**	**Recognition Test Distractors**
We Wish You a Merry Christmas	Christmas	patriotic, birthday
Take Me Out to the Ballgame	baseball game	horse racing, end of the day
First Call	horse racing	Olympics, New Year’s
NBC chimes	NBC	bedtime, circus
Taps	military funeral	romantic, presidential anthem
Bridal Chorus	wedding	presidential anthem, Christmas
Reveille	military wake up	westerns and chase scenes, wedding
Pomp and Circumstance	graduation	wedding, Olympics
Star Spangled Banner	national anthem, patriotic	wedding, congratulations
Entry of the Gladiators	circus	military wake up, graduation
Auld Lang Syne	New Year’s Eve	congratulations, national anthem
William Tell Overture	westerns and chase scenes	circus, funeral
For He's a Jolly Good Fellow	congratulations	New Year’s, westerns and chase scenes
Wedding March	wedding	national anthem, military funeral
Lullaby	bedtime	funeral, patriotic
Hail to the Chief	presidential anthem	graduation, wedding
Funeral March	funeral	end of the day, romantic
Stars and Stripes Forever	patriotic	birthday, baseball game
Bugler's Dream	Olympics	baseball game, horse racing
Happy Birthday	birthday	Christmas, military wake up

Table S3. Stimulus Items: Sports Recall and Recognition Tests

Question	Correct Response	Recognition Test Distractors
The Yankees are a professional baseball team from what city?	New York City	Boston, Philadelphia
In baseball, which player throws the ball to the batter?	pitcher	catcher, outfielder
What sport does Manchester United play?	soccer	tennis, basketball
How does each point of a tennis game start?	a serve	a forehand, a pitch
What term is used when a baseball player hits the ball and runs around all the bases to score?	homerun	around the world, skyshot
What sport did Michael Jordan play?	basketball	tennis, swimming
How many yards are between the two goal lines of a football field?	100 yards	50 yards, 250 yards
Which soccer player protects the net?	goalie	defenders, referee
How much is the first point of a tennis game worth?	15	30, 5
What term is used when a basketball player takes more than two steps without bouncing the ball?	traveling	dribbling, hiking
What is the name of the equipment used to hit a tennis ball?	racket	bat, club
In baseball there is a covered shelter along each baseline where the players go when off the field. What are these shelters called?	dugouts	bull pen, forts
What sport is played in this location? (presented with picture of basketball court)	basketball	football, ice hockey
What professional golfer was considered the best in the world from 1999-2003?	Tiger Woods	Jack Nichlaus, Arnold Palmer
What sport did John McEnroe play?	tennis	baseball, football
How many strikes result in a baseball player being out?	3	2, 5
What is the name of the equipment used to hit the ball in baseball?	bat	racket, club
What is the name of the shot in which a volleyball player forcefully drives the ball downward over the net?	spike	strike, bump
What country did the Olympics first take place in?	Greece	Italy, France
What US city do the Red Sox come from?	Boston	Baltimore, Miami
Which African American track star won four gold medals in the 1936 Berlin Summer Olympics?	Jesse Owens	Carl Lewis, Reggie White
What sport was Muhammad Ali famous for?	boxing	football, tennis
What sport do the Harlem Globetrotters play?	basketball	soccer, baseball
What sport does this athlete play? (presented with picture of football player)	football	ice hockey, tennis
What country does the Superbowl take place in?	USA	England, Spain
What two seasons do the Olympics take place in?	summer and winter	fall and spring, summer and fall
Who was the first African-American to play major league baseball in the modern era?	Jackie Robinson	John Jorgensen, Babe Ruth

What is the name of the individual who carries a golfer's bag of clubs?	caddy	greenskeeper, referee
What sport does this athlete play? (presented with picture of baseball player)	baseball	soccer, football
What country hosts the major tennis tournament Wimbledon?	England	France, Brazil
What Olympic symbol is carried to the Olympic games by a relay of runners?	torch	baton, candle
What 1960's basketball player was the first to score 100 points in a game?	Wilt Chamberlain	Dick Barnet, Kareem Abdul–Jabbar
Which golf club would you use to hit the ball the farthest?	driver	wedge, putter
What does it mean for a baseball player to be "on deck" to bat for his team?	is first in line	is second in line, is third in line
What is the defensive move called when a football player forces an opposing player to the ground?	tackle	knockdown, ace
How many holes are on a professional golf course?	18	9, 36
The MLB represents the Major League of what professional sport?	Major League Baseball	Major League Basketball, Major League Bowling
What kind of sport ball is this? (presented with picture of basketball)	basketball	soccer ball, football
What sport do the Philadelphia Flyers play?	ice hockey	football, baseball
In baseball, what infielder stands between second and third base?	shortstop	left outfielder, catcher
What country did Mia Hamm play for in the Olympics?	USA	England, Spain
Without extra innings, how many innings are in a professional baseball game?	9	7, 2
What is it called when a legal serve cannot be returned by the opponent in tennis?	ace	fault, strike
In billiards (or pool) what is it called when the white cue ball is knocked into a pocket?	scratch	re-rack, fumble
What is it called when a bowler knocks down all his pins on his first shot?	strike	spare, split
What term is used for the area in which a football player scores a touchdown?	end zone	side zone, strike zone
In the long jump, athletes jump into a pit filled with what material?	sand	pebbles, water
In tennis, a score of "zero" may also be called what?	love	null, zip
In football, how many points is a touchdown worth?	6	3, 1
What part of a standard bicycle is used to steer?	handlebars	pedals, gears
The athlete who finishes first in an Olympic event wins a gold medal. What kind of medal is awarded for third place?	bronze	copper, gold
How many points is a soccer goal worth?	1	2, 6
What is the name of the object that a golfer places the ball on before swinging?	tee	wedge, stand
What is dribbling in basketball?	bouncing the ball with your hands	holding the ball in your hands, kicking the ball with your feet
In a golf match, what does it mean when a hole is described as a Par 4?	It should only take 4 shots to put the ball in the hole	It is the 4th hardest hole in the golf course, No more than 4 people should play the hole at once

Table S4. Stimulus Items: Art Recall and Recognition Tests

**Painting**	**Artist**	**Recognition Test Distractors**
Starry Night	Vincent Van Gogh	Paul Gauguin, Sandro Botticelli
Sunrise	Claude Monet	Pierre-Auguste Renoir, Andrew Wyeth
La Danse	Henri Matisse	Paul Klee, Domenico Ghirlandaio
Garden of Earthly Delights	Hieronymus Bosch	Pieter Brueghel the Elder, Mary Cassatt
The Fighting Temeraire	Joseph Turner	Claude Monet, René Magritte
Sunday Afternoon on the Island of La Grande Jatte	Georges Seurat	Camille Pissarro, Roy Lichtenstein
Around the Fish	Paul Klee	Henri Matisse, Caravaggio
Rain, Steam, and Speed	Joseph Turner	Claude Monet, El Greco
Self Portrait with a Bandaged Ear	Vincent Van Gogh	Paul Cézanne, Titian
Sunflowers	Vincent Van Gogh	Paul Cézanne, Jan Vermeer
View of Toledo	El Greco	Francisco Goya, Mark Rothko
A Girl with a Watering Can	Pierre-Auguste Renoir	Mary Cassatt, Willem de Kooning
Birth of Venus	Sandro Botticelli	Domenico Ghirlandaio, Joan Miró
Summertime	Edward Hopper	Andrew Wyeth, Wassily Kandinsky
The Arnolfini Portrait; The Arnolfini Wedding	Jan van Eyck	Rogier van der Weyden, Max Ernst
Rouen Cathedral in Full Sunlight	Claude Monet	Pierre-Auguste Renoir, Robert Rauschenberg
Crying Girl	Roy Lichtenstein	Andy Warhol, Pierre-Auguste Renoir
The Treachery of Images	René Magritte	Marcel Duchamp, Peter Paul Rubens
Self Portrait	Pablo Picasso	Paul Cézanne, Giovanni Bellini
Lady with a Unicorn	Raphael	Leonardo da Vinci, Vincent Van Gogh
Shooting of May 3rd	Francisco Goya	Édouard Manet, Edward Hopper
Number 8	Jackson Pollock	Willem de Kooning, Georges Seurat
Guernica	Pablo Picasso	Georges Braque, Leonardo da Vinci
Luncheon of the Boating Party	Pierre-Auguste Renoir	Édouard Manet, Francisco Goya
Mona Lisa	Leonardo Da Vinci	Raphael, Georges Braque
The Son of Man	René Magritte	Salvador Dalí, Édouard Manet
Venus of Urbino	Titian	Giovanni Bellini, Marcel Duchamp
Twittering Machine	Paul Klee	Joan Miró, Paul Cézanne
The Café Terrace on the Place du Forum	Vincent Van Gogh	Paul Cézanne, Jan van Eyck
A Bar at the Folies-Bergère	Édouard Manet	Pierre-Auguste Renoir, Rogier van der Weyden
Virgin of the Rocks	Leonardo Da Vinci	Michelangelo, Grant Wood

Arrangement in Grey and Black No.1 [Whistler's Mother]	James Whistler	Andrew Wyeth, Hieronymus Bosch
Senecio	Paul Klee	Pablo Picasso, Pieter Brueghel the Elder
The Girl with the Pearl Earring	Jan Vermeer	Caravaggio, Jackson Pollock
Where Do We Come From? What Are We? Where Are We Going?	Paul Gauguin	Pablo Picasso, Diego Velázquez
The Scream	Edvard Munch	Vincent Van Gogh, Raphael
Light Iris	Georgia O'Keeffe	Claude Monet, Edvard Munch
The Creation of Adam	Michelangelo	Leonardo da Vinci, Camille Pissarro
Jazz: The Horse, the Horsewoman and the Clown	Henri Matisse	Paul Klee, Claude Monet
Girl with Red Beret	Pablo Picasso	Paul Gauguin, Michelangelo
Bridge Over a Pool of Water Lilies	Claude Monet	Pierre-Auguste Renoir, Pablo Picasso
Venus at the Mirror	Peter Paul Rubens	Titian, Vincent Van Gogh
Human Condition or Promenades of Euclid	René Magritte	Max Ernst, Georgia O'Keeffe
The Card Players	Paul Cézanne	Vincent Van Gogh, Salvador Dalí
The Circus	Georges Seurat	Pierre-Auguste Renoir, Frans Hals
Van Gogh's Room at Arles	Vincent Van Gogh	Henri de Toulouse-Lautrec, James Whistler
The Dancing Class	Edgar Degas	Édouard Manet, Paul Klee
Las Meninas	Diego Velázquez	Francisco Goya, Maxfield Parrish
Upside Down Figures	Joan Miró	Wassily Kandinsky, Édouard Manet
Goldfish	Henri Matisse	Vincent Van Gogh, Leonardo da Vinci
Three Musicians	Pablo Picasso	Georges Braque, Paul Gauguin
Apples and Oranges	Paul Cézanne	Paul Gauguin, Andy Warhol
The Persistence of Memory	Salvador Dalí	Max Ernst, Claude Monet
The Kiss	Gustav Klimt	Maxfield Parrish, Joseph Turner
Red Studio	Henri Matisse	Paul Klee, Edgar Degas
Self Portrait	Rembrandt	Frans Hals, Gustav Klimt
The Dane in the Moulin Rouge	Henri de Toulouse-Lautrec	Édouard Manet, Paul Klee
Last Supper	Leonardo da Vinci	Michelangelo, Henri Matisse
New Harmony	Paul Klee	Mark Rothko, Pierre-Auguste Renoir
American Gothic	Grant Wood	Andrew Wyeth, Pablo Picasso
Campbell's Soup Cans	Andy Warhol	Robert Rauschenberg, Paul Cézanne
Self Portrait	Paul Gauguin	Henri Matisse, Rembrandt
The Last Judgment	Michelangelo	El Greco, Henri de Toulouse-Lautrec

Table S5. Stimulus Items: Classical Music Recall and Recognition Tests

Classical Music Piece	Composer	Recognition Test Distractors
Bolero	Maurice Ravel	Georges Bizet, Johann Pachelbel
Rhapsody in Blue	George Gershwin	Leonard Bernstein, Ludwig van Beethoven
Canon in D	Johann Pachelbel	Johann Sebastian Bach, Gustav Mahler
Piano Concerto No. 2	Sergei Rachmaninoff	Pyotr Ilyich Tchaikovsky, John Williams
Ride of the Valkyries	Richard Wagner	Richard Strauss, Maurice Ravel
Minuet in G Major	Johann Sebastian Bach	Wolfgang Amadeus Mozart, John Philip Sousa
The Planets: Mars	Gustav Holst	John Williams, Edvard Grieg
Requiem: Lacrimosa	Wolfgang Amadeus Mozart	George Frideric Handel, Philip Glass
The Firebird Suite: Finale	Igor Stravinsky	Ottorino Respighi, George Frideric Handel
Symphony No. 9: "Ode to Joy"	Ludwig van Beethoven	George Frideric Handel, Georges Bizet
The Tale of Tsar Saltan: The Flight of the Bumblebee	Nikolai Rimksy-Korsakov	Sergei Rachmaninoff, Dave Brubeck
Swan Lake	Pyotr Ilyich Tchaikovsky	Camille Saint-Saëns, Gioachino Rossini
The Marriage of Figaro: Overture	Wolfgang Amadeus Mozart	Gioachino Rossini, Vince Guaraldi
Water Music Suite in D Major: Alla Hornpipe	George Frideric Handel	Johann Sebastian Bach, Edward Elgar
Orpheus in the Underworld: Infernal Gallop	Jacques Offenbach	Gioachino Rossini, Claude Debussy
Pomp and Circumstance	Edward Elgar	Felix Mendelssohn, Gioachino Rossini
Jesu: The Joy of Man's Desiring	Johann Sebastian Bach	Johann Pachelbel, Carl Orff
The Four Seasons: Spring	Antonio Vivaldi	George Frideric Handel, Frédéric Chopin
The Rite of Spring: Sacrificial Dance	Igor Stravinsky	Dmitri Shostakovich, Pyotr Ilyich Tchaikovsky
The Sleeping Beauty: Garland Waltz	Pyotr Ilyich Tchaikovsky	Johann Strauss, Wolfgang Amadeus Mozart
Fanfare for the Common Man	Aaron Copland	John Williams, Scott Joplin
Piano Concerto in A minor	Edvard Grieg	Pyotr Ilyich Tchaikovsky, Henry Mancini
The Carnival of the Animals: The Swan	Camille Saint-Saëns	Franz Liszt, Jimmy Dorsey
The Magic Flute: Queen of the Night	Wolfgang Amadeus Mozart	Gioachino Rossini, Ottorino Respighi
A Midsummer Night's Dream: Wedding March	Felix Mendelssohn	Richard Wagner, Nikolai Rimksy-Korsakov
Maple Leaf Rag	Scott Joplin	Joseph Lamb, Ludwig van Beethoven
Claire de Lune	Claude Debussy	Philip Glass, Frédéric Chopin
Brandenburg Concerto no. 3	Johann Sebastian Bach	George Frideric Handel, Aaron Copland
Peter and the Wolf: The Story Begins	Sergei Prokofiev	Edvard Grieg, Robert Schumann
Baby Elephant Walk	Henry Mancini	Dave Brubeck, Johann Sebastian Bach

Symphony no. 5	Ludwig van Beethoven	Felix Mendelssohn, George Gershwin
1812 Overture	Pyotr Ilyich Tchaikovsky	Gioachino Rossini, George Frideric Handel
Peer Gynt Suite No. 1: Morning Mood	Edvard Grieg	Sergei Prokofiev, Ennio Morricone
Blue Danube Waltz	Johann Strauss	Pyotr Ilyich Tchaikovsky, George Frideric Handel
The Entertainer	Scott Joplin	James Scott, Richard Strauss
Toccata and Fugue	Johann Sebastian Bach	Wolfgang Amadeus Mozart, Hoagy Carmichael
Funeral March	Frédéric Chopin	Ludwig van Beethoven, Antonín Dvořák
Moonlight Serenade	Glenn Miller	Jimmy Dorsey, Johannes Brahms
Cello Suite 1 in G Major	Johann Sebastian Bach	Ludwig van Beethoven, Jacques Offenbach
Piano Sonata No. 8 in C Minor "Pathetique"	Ludwig van Beethoven	Wolfgang Amadeus Mozart, Richard Wagner
Carmen: Les Toreadors	Georges Bizet	Gioachino Rossini, Joseph Lamb
Carmina Burana: O Fortuna	Carl Orff	Gustav Mahler, Pyotr Ilyich Tchaikovsky
Also Sprach Zarathustra	Richard Strauss	John Williams, Franz Schubert
Marche Slave	Pyotr Ilyich Tchaikovsky	Antonín Dvořák, Joseph Hadyn
Symphony No. 9 "New World"	Antonín Dvořák	Richard Wagner, Wolfgang Amadeus Mozart
Eine Kleine Nachtmusik	Wolfgang Amadeus Mozart	Johann Sebastian Bach, Dmitri Shostakovich
Brahms Lullaby	Johannes Brahms	Franz Schubert, James Scott
Symphony No. 40	Wolfgang Amadeus Mozart	Felix Mendelssohn, Richard Wagner
Bagatelle No. 25 in A Minor "Fur Elise"	Ludwig van Beethoven	Franz Liszt, Igor Stravinsky
Piano Sonata No. 11 in A Major: Alla Turca	Wolfgang Amadeus Mozart	Joseph Hadyn, Glenn Miller
The Barber of Seville: Overture	Gioachino Rossini	Wolfgang Amadeus Mozart, Sergei Rachmaninoff
Linus and Lucy	Vince Guaraldi	Hoagy Carmichael, Camille Saint-Saëns
The Four Seasons: Winter	Antonio Vivaldi	Wolfgang Amadeus Mozart, Franz Liszt
The Stars and Stripes Forever	John Philip Sousa	Johann Strauss, Felix Mendelssohn
Orchestral Suite no. 2 in B minor: Badinerie	Johann Sebastian Bach	Antonio Vivaldi, Johann Strauss
Carmen: Habanera	Georges Bizet	Maurice Ravel, Antonio Vivaldi
Rodeo: Hoedown	Aaron Copland	Ennio Morricone, Wolfgang Amadeus Mozart

Table S6. Stimulus Items: United States History Recall Test

Question	Sample Acceptable Responses
What is one right or freedom from the 1st Amendment?	Speech, religion, assembly, press, petition the government
What is the economic system in the US?	Capitalist economy, market economy
Name one or more branches or parts of the government.	Congress, legislative, president, executive, the courts, judicial
What are the two parts of the US Congress?	Senate and House of Representatives
Who is one of your state's U.S. Senators now?	[Acceptable response varied with state of residence]
In what month do we vote for President?	November
What is the name of the President of the United States now?	Barack Obama
What is the capital of your state?	[Acceptable response varied with state of residence]
What are the two major political parties in the United States?	Democratic and Republican
What is one responsibility that is only for United States citizens?	serve on a jury, vote in a federal election
How old do citizens have to be to vote for President?	18 and older
When is the last day you can send in federal income tax forms?	April 15
Who was the first President?	George Washington
What important thing(s) did Abraham Lincoln do?	freed the slaves (Emancipation Proclamation, saved (or preserved) Union, led the US during the Civil War
Name one or more wars fought by the United States in the 1900s.	WWI, WWII, Korean War, Vietnam War, (Persian) Gulf War
What did Martin Luther King, Jr. do?	fought for civil rights, worked for equality for all Americans
What is the capital of the United States?	Washington, D.C.
Where is the Statue of Liberty?	New York (Harbor), Liberty Island, Also OK are NJ, near NYC, on the Hudson (River)
Why does the flag have 50 stars?	there is one star for each state, each star represents a state, there are 50 states
When do we celebrate Independence Day?	July 4
Why did colonists come to America?	freedom, political liberty, religious freedom, economic opportunity, practice their religion, escape persecution
Who lived in American before the Europeans arrived?	American Indians, Native Americans
What group of people was taken to America and sold as slaves?	Africans, people from Africa

Why did the colonists fight the British?	high taxes (taxation w/o representation), the British army stayed in their houses (boarding, quartering), they didn't have self-government
Who wrote the Declaration of Independence?	Thomas Jefferson
When was the Declaration of Independence adopted?	July 4, 1776
There were 13 original states. Can you name some?	New Hampshire, Massachusetts, Rhode Island, Connecticut, New York, New Jersey, Pennsylvania, Delaware, Maryland, Virginia, North Carolina, South Carolina, Georgia
What happened at the Constitutional Convention?	The Constitution was written, the Founding Fathers wrote the Constitution
When was the Constitution written?	1787
The Federalist Papers supported the passage of the U.S. Constitution. Who were the [illegible]	James Madison, Alexander Hamilton, John Jay, Publius (pseudonym used by authors)
What is Benjamin Franklin famous for?	U.S. diplomat, oldest member of the Constitutional Convention, first Postmaster General of the US, writer of "Poor Richard's Almanac", started the first free libraries
Who is the "Father of Our Country"?	George Washington
What territory did the United States buy from France in 1803?	the Louisiana Territory, Louisiana
Name one or more wars fought by the United States in the 1800s.	War of 1812, Mexican-American War, Civil War, Spanish-American War
Name the U.S. war between the North and the South.	the Civil War, the War between the States
Name one or more problems that led to the Civil War.	slavery, economic reasons, states' rights
What did the Emancipation Proclamation do?	freed the slaves, freed slaves in the Confederacy/the Confederate states/most Southern states
What did Susan B. Anthony do?	fought for women's rights, fought for civil rights
Who was President during World War I?	Woodrow Wilson
Who was President during the Great Depression and World War II?	Franklin Roosevelt
Who did the United States fight in World War II?	Japan, Germany, and Italy
Before he was President, Eisenhower was a general. What war was he in?	World War II
During the Cold War, what was the main concern of the United States?	Communism
What movement tried to end racial discrimination?	civil rights (movement)
What major event happened on September 11, 2001, in the United States?	Terrorists attacked the United States
Name one or more American Indian tribes in the United States.	Cherokee, Navajo, Sioux, Chippewa, Choctaw, Pueblo, Apache, Iroquois, Creek, Blackfeet, Seminole, Cheyenne, Arawak

Table S7. Stimulus Items: United States History Recognition Test

Famous Individual	President?
Douglas MacArthur	N
Thurgood Marshall	N
John Tyler	Y
Orville Wright	N
George H.W. Bush	Y
John Quincy Adams	Y
Thomas Edison	N
William Lloyd Garrison	N
Frederick Douglass	N
James Fenimore Cooper	N
George W. Bush	Y
Benjamin Harrison	Y
Woodrow Wilson	Y
James Madison	Y
Lyndon B. Johnson	Y
J.P. Morgan	N
James K. Polk	Y
James A. Garfield	Y
John C. Calhoun	N
Henry David Thoreau	N
William Randolph Hearst	N
James Monroe	Y
William Henry Harrison	Y
Grover Cleveland	Y
Oliver Wendell Holmes	N
Earl Warren	N
Franklin D. Roosevelt	Y
Rutherford B. Hayes	Y
Zachary Taylor	Y
Charles Lindbergh	N
Robert E. Lee	N
Brigham Young	N
Benjamin Franklin	N
Martin Van Buren	Y
Jimmy Carter	Y
Thomas Jefferson	Y
Richard Nixon	Y
Walter Mondale	N
Hubert H. Humphrey	N
Benedict Arnold	N
Harry S. Truman	Y
Andrew Carnegie	N

Herbert C. Hoover	Y
Walter Cronkite	N
Alexander Hamilton	N
Thomas Paine	N
Mark Twain	N
Ulysses S. Grant	Y
John Wilkes Booth	N
Theodore Roosevelt	Y
Walt Whitman	N
Warren G. Harding	Y
Andrew Jackson	Y
Bill Clinton	Y
Bob Dole	N
William McKinley	Y
William Howard Taft	Y
Horace Mann	N
Dwight D. Eisenhower	Y
Franklin Pierce	Y
William Jennings Bryan	N
John Dewey	N
Francis Scott Key	N
Calvin Coolidge	Y
Stephen Foster	N
Chester A. Arthur	Y
John D. Rockefeller	N
Gerald Ford	Y
Frank Lloyd Wright	N
Ralph Waldo Emerson	N
Abraham Lincoln	Y
Cyrus McCormick	N
Barack Obama	Y
James Buchanan	Y
Adlai Stevenson	N
Daniel Boone	N
George Washington	Y
Bill Gates	N
Henry Clay	N
George Patton	N
John Adams	Y
John F. Kennedy	Y
Andrew Johnson	Y
Louis Sullivan	N
Ronald Reagan	Y
Millard Fillmore	Y
